# A workplace Acceptance and Commitment Therapy (ACT) intervention for improving healthcare staff psychological distress: A randomised controlled trial

**DOI:** 10.1371/journal.pone.0266357

**Published:** 2022-04-20

**Authors:** Arianna Prudenzi, Christopher D. Graham, Paul E. Flaxman, Sarah Wilding, Fiona Day, Daryl B. O’Connor

**Affiliations:** 1 School of Psychology, University of Leeds, Leeds, United Kingdom; 2 School of Psychology, Queen’s University Belfast, Belfast, Northern Ireland, United Kingdom; 3 Department of Psychology, University of London, London, United Kingdom; 4 Fiona Day Consulting LTD, Leeds, United Kingdom; University of Jyvaskyla, FINLAND

## Abstract

The levels of psychological distress and burnout among healthcare staff are high, with negative implications for patient care. A growing body of evidence indicates that workplace programmes based on Acceptance and Commitment Therapy (ACT) are effective for improving employees’ general psychological health. However, there is a paucity of research examining the specific psychological and/or behavioural processes through which workplace ACT programmes transmit their beneficial effects. The aim of this randomised controlled trial was to investigate the outcomes and putative processes of change in a 4-session ACT training programme designed to reduce psychological distress among healthcare staff (n = 98). Ninety-eight employees of a healthcare organisation were randomly allocated to the ACT intervention or to a waiting list control group. Study measures were administered on four occasions (baseline, mid-intervention, post-intervention, and follow-up) over a three-month evaluation period. Results showed that the ACT intervention led to a significant decrease in symptoms of psychological distress and a less pronounced reduction in burnout. These effects were mediated primarily via an improvement in mindfulness skills and values-based behaviour and moderated by participants’ initial levels of distress. At four-week post-intervention, 48% of participants who received the ACT intervention showed reliable improvements in psychological distress, with just under half of the aforementioned improvements (46.15%) meeting criteria for clinically significant change. The results advance ACT as an effective stress management intervention for healthcare staff. The findings should be confirmed in a large scale randomised controlled trial with longer follow-up and cost-effectiveness analyses.

## Introduction

High levels of burnout and distress are a long-standing concern in the healthcare workforce [1]. For example, in the United Kingdom’s National Health Service (NHS), the estimated proportion of healthcare professionals (HCPs) reporting work-related stress has risen from 28% to 37% since 2008, with more than 25% of staff in this sector experiencing symptomatic levels of psychological distress [2]. Moreover, high levels of burnout and distress may have implications for the quality of patient care, potentially leading to reduced perceptions of patient safety and heightened risk of medical error [3, 4].

Evidence suggests that worksite stress management interventions, particularly those based on Cognitive Behaviour Therapies (CBT), can be effective for improving the psychological health of staff working in healthcare and other organisational settings [5–8]. Stress management interventions can be broadly classified into primary, secondary, or tertiary prevention initiatives [9]. Primary interventions aim to modify sources of work-related stress (e.g., lack of workplace support, feedback, or job control), while secondary interventions, such as CBT and mindfulness-based interventions, equip employees with psychological skills and resources that increase resilience to stress [10]. Tertiary interventions are considered more therapeutic in nature and are designed to mitigate clinically relevant levels of psychological distress.

### Acceptance and Commitment Therapy in the workplace

To prevent and reduce distress among employees, researchers have begun implementing and evaluating mindfulness-based worksite training interventions. One of these interventions is based on Acceptance and Commitment Therapy (ACT) [11]. Among the modern CBT approaches, ACT is increasingly being adapted for delivery to groups of employees in healthcare and other workplace settings [12–14]. Moreover, ACT interventions have also been found to lead to improvements in various other indicators of employee wellbeing and performance including increasing innovation in the workplace [12, 15, 16]. Whether delivered in clinical or nonclinical contexts, ACT interventions are designed to help people develop a broad capacity labelled psychological flexibility. Psychological flexibility is defined as “the ability to persist or to change behaviour in a setting of competing psychological influences, guided by values and goals dependent on what the situation at hand affords” [17]. In workplace settings, this ability may involve: 1) greater willingness to experience unpleasant thoughts and emotions that arise while performing at work; 2) an ability to bring present moment awareness to current tasks, inner experiences, and external interactions and situations; and 3) consistent engagement in work-related behaviours that are congruent with one’s overarching goals and values [13, 18, 19]. Psychological flexibility is developed through a combination of processes, such as mindfulness, cognitive defusion, the use of metaphor, personal values clarification, and values-based behavioural activation [20]. Higher levels of psychological flexibility have been shown to be associated with improved mental health, job performance and absenteeism in UK workers [21, 22].

When applied in the workplace, ACT most commonly involves group skills training sessions, or (less commonly) remote bibliotherapy (e.g., giving participants self-help books) or online programmes. To date, ACT interventions have been empirically evaluated in a wide range of organisational contexts, including a media organisation [15], government agencies [12, 19, 23], and in educational, social care, and healthcare settings [13, 14, 24–29]. A few studies have demonstrated the efficacy of self-help or web-based ACT programmes delivered to mixed occupational samples [30, 31]. Collectively, the results of this body of intervention research suggest that increasing psychological flexibility via relatively brief workplace ACT programmes leads to improvements in employees’ general mental health [15, 25], while the impact of ACT on burnout has been less consistent [13]. In only a small proportion of workplace studies, ACT has been found to improve performance-related outcomes [15, 16].

### Why ACT interventions in the workplace?

Despite the burgeoning popularity of ACT in workplace settings, and the accumulating evidence supporting ACT’s utility for improving employees’ general mental health, a number of issues remain contentious or underexplored. First, workplace evaluations of ACT have focused predominantly on broad process of change variables, particularly overall levels of psychological flexibility and/or mindfulness [13, 19, 25]. Generally, this research does not determine the more discrete psychological and/or behavioural skills being developed by employees who attend ACT programmes; nor does it establish whether a subset of the targeted skills are especially influential in transmitting ACT’s positive effects on employees’ mental health.

With these potential theoretical and practical benefits in mind, the first contribution of the current trial is to ascertain the degree to which an ACT programme delivered to staff in a healthcare setting elicits change on a set of psychological flexibility’s sub-processes (e.g., mindfulness, values and self-compassion); and whether change on some or all of these processes mediates ACT’s effect on healthcare employees’ mental health.

Our second contribution is to address the inconsistent findings surrounding the putative impact of ACT on key aspects of work-related functioning, beginning with job burnout [13]. As theorised by the Job Demands-Resources Model, burnout may stem largely from chronic work environment conditions, such as excessive job demands (e.g., work overload and time pressure) coupled with a lack of job resources (e.g., support, autonomy, or feedback) [32]. Research evaluating ACT for burnout has mostly utilised the Maslach Burnout Inventory [13, 14]. Although the Maslach Burnout Inventory is widely used in the burnout literature, it is noteworthy that the original scale instructions ask employees to report burnout symptoms experienced over a wide timeframe (e.g., a few times per year or less up to every day). In contrast, worksite ACT evaluations tend to be conducted over comparatively shorter periods (e.g., 3 months). This incongruence between measurement and study design may reduce the likelihood of detecting longer-term changes in burnout elicited by ACT programmes.

Consistent with these principles, the Conservation of Resources theory [33, 34] conceptualises burnout as an over-depletion of energy resources resulting from prolonged exposure to job stress without adequate periods of recovery. On the basis of these observations, we propose that the Conservation of Resources theory conceptualisation and assessment is well-suited to the task of exploring shorter-term changes in healthcare workers’ burnout symptoms elicited by a workplace ACT intervention. An additional advantage of drawing from this theory is that it supports assessment of change on other variables that are theoretical and empirically associated with burnout. A well-established risk factor for burnout is lack of recovery from work demands during non-work time (e.g., post work evenings, weekends, or non-shift days) [35]. To our knowledge, no previous research has explored whether attending an ACT programme strengthens employees’ ability to cognitively detach from work problems during non-work time.

The third contribution is to extend the Conservation of Resources theory to examine ACT’s influence on a primary indicator of work-related functioning in healthcare settings: perceptions of patient safety [3]. Given the critical importance of safe practice in healthcare contexts [3], we examine whether any salutary effect of ACT on healthcare employees’ exhaustion is associated with an improvement in their perceptions of patient safety. In this way, we respond to calls for worksite ACT research to incorporate performance-related outcomes [13], and assess whether the growing uptake of ACT for improving healthcare staff wellbeing might translate into improvements in the quality of patient care.

Lastly, because these programmes are typically offered and delivered in practice to all employees, regardless of current levels of psychological strain, with the fourth contribution we aim to evaluate this programme in the same way it would likely be delivered in other healthcare organisations. Because ACT has increasingly being found to be useful for employees with above average levels of psychological strain [19, 29, 30], we also aim to test whether initial level of strain moderates the effects. If initial level of psychological strain moderates the intervention effects, this study would provide useful information to organisations who may consider whether to offer ACT widely, or specifically seek to target employees with high levels of strain (i.e., it has implications for how the programmes are delivered within the NHS).

### The present study

Based upon the current evidence and the need to further test whether ACT interventions are helpful and effective for improving mental health in healthcare professionals, we investigated whether a shorter version of the validated and standardised intervention was effective for improving psychological distress and burnout in NHS staff. Unlike the traditional version of the intervention with three sessions carried out across three months, the current intervention was delivered in four sessions across one month. Also, for the first time, the current study aimed to assess whether the intervention could have a direct or indirect impact on perceived patient safety.

Based on the rationale outlined above, we tested the following five hypotheses:

Hypothesis 1: Employees who attend a workplace ACT intervention will experience a significant improvement in mental health, specifically a reduction in symptoms of psychological distress (primary outcome) along with reductions in burnout, work-related worry and rumination and perceived patient safety (secondary outcomes)
Hypothesis 2: Positive effects of the ACT intervention on outcome variables will be mediated through improvements in psychological flexibility processes.
Hypothesis 3: ACT intervention effects will be moderated by initial levels of psychological distress.
Hypothesis 4: The proportion of participants reporting reliable and clinically significant changes in psychological distress at post-intervention and follow-up will be greater in the intervention group compared to the waitlist control group.
Hypothesis 5: The ACT intervention will be associated with an improvement in perceived patient safety.

## Materials and method

The study received ethical approval by the Health Research Authority R&D approval (IRAS ref#18/HRA/0200 accepted on 21/09/2017) and the School of Psychology’s Research Ethics Committee (ref#17–0212 accepted on 22/07/2017). The study protocol was registered in the ISRCTN registry (ISRCTN29599982 accepted on 20/07/2018). Note that hypotheses 1 and 2 were preregistered as part of the study protocol and hypotheses 3–5 were not and therefore, are considered to be exploratory.

The reporting of this psychological trial is in line with the Consolidated Standards of Reporting Trials (CONSORT) guidelines [36]. The study was registered after enrolment of participants started but before data collection due to an unexpected delay as a result of a change in personnel related to conducting the trial.

Data of this study are available at the Open Science Framework (OSF): DOI 10.17605/OSF.IO/ZNCWA.

### Design

The study employed a parallel group randomised controlled design.

### Participants

Eligible participants were currently employed NHS staff working within NHS primary care settings in Yorkshire and Humberside. Participants were excluded if not present at work. Based on previous RCT studies of ACT interventions in HCPs [24, 29], we estimated a small to medium effect size (*f* = 0.22). The estimated sample size necessary to detect a small to medium effect size at an alpha rate of 0.05 (two-tailed) using the G* Power 3 programme [37], was 104 participants. However, based on the average drop-out rate reported in previous ACT interventions for HCPs [12, 24, 29], we aimed to receive consent from up to 140 participants, anticipating approximately a 26% attrition rate.

**Fig 1:**
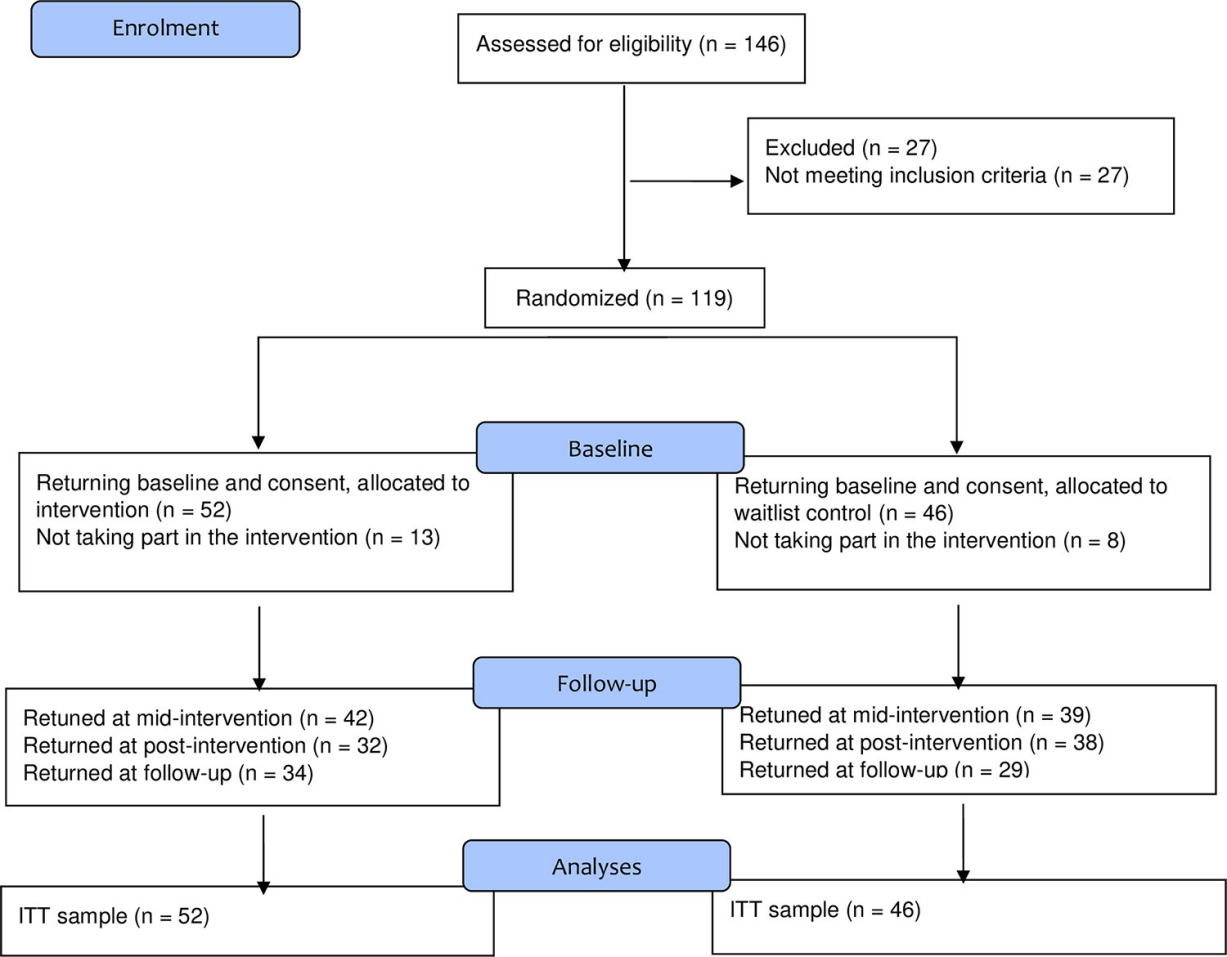
Participants flow.

A total of 146 NHS staff returned the consent form. 98 NHS staff (52 experimental, 46 controls) were finally included in the study, see CONSORT flowchart in Fig 1. 81 participants completed measures at mid-intervention (17.3% attrition rate), 70 participants responding at post-intervention (28.6% attrition rates) and 63 at follow-up (35.8%attrition rate). The mean age of participants was 42.97 years old (SD = 10.18). 92.7% of the participants were female. Participants worked 34.14 hours per week on average (SD = 7.59), and 46.3% worked full-time. Participants were from a range of NHS roles: GPs (28%), nurses (23.2%), mental health professionals (17.1%), managers (13.4%), administration staff (7.3%), consultants (2.4%), dietitians (2.4%), other HCPs (1.2%), technicians (2.4%), other staff (2.4%). 87.8% of the sample was English, Welsh, Scottish or Northern Irish, 2.4% was European, and the remainder (1.2%) was Brazilian, Caribbean, Indian, Latin American, Pakistani, or White Chinese. Based on 83.7% of the sample reporting demographics, 14.3% of the sample reported practicing mindfulness at baseline, 6.1% by using an app, 4% reported doing meditation, 2% yoga and a final 2% practicing stress-management exercises. 56.1% of the sample did not report practicing mindfulness. The frequency of mindfulness practice of those who were practicing mindfulness at baseline was weekly (12.2%), daily (7.1%), monthly (5.1%), two-three times per week (4.1%), ad hoc (1%).

### Procedure

Potential participants were contacted via posters, emails and local awareness via primary care settings. The ACT training was open to NHS staff currently at work. Participants were given information about the study and contacts of primary care managers if interested in opting-in to the study. The participant information sheet, with a briefing about the study and training, was sent via e-mail to potentially interested participants. After providing consent, participants were randomly assigned to an experimental group (receiving the ACT intervention) or a waitlist control group. A computer-generated randomisation procedure was employed. Participants allocated to the waitlist control were informed that they would receive the intervention after fourteen weeks.

The training programme was delivered in experimental or waitlist cohorts from October 2017 to October 2018. For example, if a participant was randomised to the experimental arm late September 2017, the participant took part in the intervention from October 2017 to January 2018. Instead, the waitlist control group participant (enrolled in the waitlist control arm) would have completed the intervention from January to March 2018. Participants allocated to the intervention or waitlist control groups completed outcomes and process measures at baseline (i.e. up to four weeks before the start of the intervention), at mid-intervention (after session 2, and between week 1 and 3 from the start of the intervention), at four-week after the start of the intervention (at the end of the four-week intervention), and at fourteen weeks follow-up (ten weeks after the end of the intervention).

The training was delivered by three mindfulness trainers (see therapists section below) to NHS employees on four different occasions (7–9 pm), during non-working hours, in North and South Leeds in venues outside the organisation. Participants received a total of 8-hour intervention time spanning across four consecutive weeks.

### ACT intervention

The ACT intervention was based on an existing standardised and validated ACT three-week intervention designed for workplace settings [38]. The current intervention was an updated version of the three half-day sessions previously utilised in workplace contexts. The intervention was extended to a total of four two-hour group sessions delivered on four consecutive weeks. Sessions comprised between 10 and 15 people. Each session consisted of a structured combination of mindfulness and value-based work.

The first session served the purpose of introducing participants to mindfulness and value-based activities. In this session, participants were introduced to both mindfulness and values psychoeducation to help them become familiar with psychological flexibility processes. Two main mindfulness-based exercises were given: the raisin exercise [39] and the body and breath meditation [40]. In terms of values, the participants were invited to a “values card sort exercise [41]. During this session, participants were also invited to reflect on barriers, thoughts and feelings that may interfere with personally valued action. The “passengers on the bus” metaphor [11] was proposed to help participants understand their psychological barriers. In every session, the trainers would invite participants to practice the exercises learned (e.g., mindfulness and value-based exercises) in their home practice. However, data on the frequency of these activities were not collected. The same structure of the first session was then repeated in the following sessions.

In the second session, participants were invited for the first time to the psychological flexibility component of cognitive defusion. After a repetition of the body and breath meditation given in session 1 [40], participants were introduced to the distinction between thinking and sensing modes of mind [42]. Next, trainers presented a retrospective noticing exercise based on a simplified version of the ACT matrix [43, 44]. During this exercise participants were invited to notice the barriers that block them from the qualities they wanted to express most. Participants were then invited to repeat the ACT matrix by focusing on values. To conclude session 2, the participants were invited to an adapted version of the “passengers on the bus metaphor” presented in session 1 but with the aim of cultivating cognitive defusion.

In the third session, participants were invited for the first time to the psychological flexibility component of acceptance. First, the trainer would start with a mindfulness exercise with the aim of helping participants bring attention to body sensations. This exercise aimed to reconnect HCPs with the body and to develop curiosity. Following the home practice review, the trainer re-proposed the retrospective noticing exercise linking it with the physicalising exercise. This exercise offered a possibility to practice acceptance. During this session the trainer read the Rumi guest house poem [44], and invited participants to repeat the ACT matrix exercise in different life areas. This session ended with a three-step mindfulness exercise.

The final session followed the structure of the previous sessions with the overall aim to bring together and refresh all psychological skills learned in the previous sessions. After the opening mindfulness exercise, the participants were invited to the home practice review, and to repeat the retrospective noticing exercise. A different version of the value-based construction exercise was repeated. The main focus of this last session was to invite the participants to participate in a role-play by acting out the “passengers on the bus” metaphor. The main purpose of this metaphor was to help participants relate to different thoughts and emotions. For a detailed description of the intervention content see S1 File.

### Measures

#### Outcome measures

General Health Questionnaire (GHQ-12) [45]. The GHQ-12 is a 12-item well-known measure of psychological distress and assesses a number of different aspects related to one’s wellbeing (e.g., feeling unhappy, depressed and constantly under strain). The GHQ-12 was scored in two ways. The Likert method (0-1-2-3) was used to assess primary outcomes. Higher scores indicate higher levels of psychological distress (1 = better than usual, 4 = much less than usual).

The scoring method (0-0-1-1) was instead employed to assess reliable and clinically significant changes. Unlike the Likert method, the scoring method is commonly used to assess clinically symptomatic levels of psychological distress. By using the scoring method, participants were classified as: asymptomatic, sub-clinically symptomatic, symptomatic, and highly symptomatic. In the present sample Cronbach’s alphas were .86 at baseline, .88 at mid-intervention, .90 at post-intervention and .90 at follow-up.

Shirom-Melamed Burnout Measure (SMBM) [46]. The SMBM is a measure of job burnout, composed of 14 items and three subscales that capture three core components of burnout (physical fatigue, emotional exhaustion, and cognitive weariness). The emotional exhaustion component of burnout measures the perception of the "drying up" of one’s emotional resources and the feeling that one has nothing to give on a psychological level (e.g., "I feel I am unable to be sensitive to the needs of colleagues and patients"). The physical component of burnout measures the physical exhaustion derived from the chronic stress (e.g., “I have no energy for going to work in the morning”). The cognitive weariness of burnout is concerned with difficulties in concentration, rigid cognitive style, dysfunctional thoughts (e.g., worries and ruminations, negative thoughts, e.g., “I have difficulty concentrating”). Higher scores indicate higher levels of burnout. The participants were asked to report how they felt at work on a scale from 1 (never felt this way at work) to 7 (always felt this way at work). Unlike other widely used measures of burnout, this scale assesses the cognitive component of burnout and is anchored to the past 30 workdays. In the present sample Cronbach’s alphas were .91 at baseline, .91 at mid-intervention, .94 at post-intervention and .94 at follow-up.

Work-related worry and rumination was assessed using two scales: the Affective Rumination Scale [47], and the Perseverative Cognition Scale [48]. The first scale [47] captures employees’ experiences of negative affectF (e.g., intrusive and/or annoying) ruminative thoughts about work during non-work time, and has demonstrated good psychometric properties among various samples of UK workers [47, 49]. This scale asked people to describe different feelings they may have at work from 1 (very seldom to never) to 5 (very often or always). The second scale was developed and validated by Flaxman and colleagues [48] It assesses the amount of work and rumination thoughts experienced during non-work time (e. g., evenings and weekends) over the past week from 1 (not at all) to 5 (a great deal). A total worry and rumination score was created by summing the scores from each scale. Higher scores indicate higher levels of worry and rumination. In the present sample Cronbach’s alphas were .90 at baseline, .91 at mid-intervention, .93 at post-intervention and .90 at follow-up.

Safe Practitioner measure [50]. This measure captures perceptions of organisational and personal patient safety and was measured using the following two items “In the past four weeks, my practice is not as safe as it could be because of work related factors/conditions” (individual perceptions of organisational safety practices) and “My practice is safe” (individual perceptions of personal safety practices), rated from 1 (strongly disagree) to 5 (strongly agree). This measure has been shown to be reliable and valid in HCPs [50, 51].

#### Process measures

In order to investigate the mechanisms of action that the ACT intervention may change, we included the following assessments of ACT process measures.

Multidimensional Experiential Avoidance Questionnaire–Distress Endurance subscale (MEAQ-DE) [52]. This 11-item MEAQ-DE subscale measures an important aspect of psychological flexibility: one’s ability to endure difficult thoughts and feelings in order to complete important activities. Higher scores indicate higher distress endurance. Items were rated on a scale that extended from 1 (strongly disagree) to 5 (strongly agree). In the present sample Cronbach’s alphas were .89 at baseline, .89 at mid-intervention, .90 at post-intervention and .87 at follow-up.

Short-Form Five Facet Mindfulness Questionnaire [53]. This is the short scale of the well-known multidimensional Five Facet Mindfulness Questionnaire Scale (51). This scale assesses 5 subcomponents of mindfulness: observing, describing, acting with awareness, non-judging of experience, and non-reactivity to difficult inner experience. In this study the total score was employed. Higher scores indicate higher levels of mindfulness from 1 (never true) to 5 (always true). In the present sample Cronbach’s alphas were .81 at baseline, .83 at mid-intervention, .86 at post-intervention and .85 at follow-up.

Valuing Questionnaire [54]. This 10-item questionnaire assesses people’s ability to engage in valuing actions. The scale has previously demonstrated good psychometric properties in both clinical and nonclinical adult populations (52). Higher scores indicate higher scores in value-based living from. Items were rated on a scale that extended from 1 (not at all true) to 6 (completely true). In the present sample Cronbach’s alphas for values progression were .74 at baseline, .84 at mid-intervention, .85 at post-intervention and .81 at follow-up. In the present sample Cronbach’s alphas for values obstruction were .80 at baseline, .80 at mid-intervention, .80 at post-intervention and .78 at follow-up.

Self-Compassion Scale–short-form [55]. The measure captures the propensity to treat oneself with care and kindness, to accept one’s imperfections, and tendency to take a balanced perspective on one’s experiences. This 12-item self-compassion scale has 6 subscales: self-kindness; self-judgement; common humanity; isolation; mindfulness; and over-identification. Higher scores indicate higher levels of self-compassion. Items were rated on a scale that extended from 1 (almost never) to 5 (almost always). The total score was employed in this study. The scale has previously demonstrated good psychometric properties in HCPs. In the present sample Cronbach’s alphas were .83 at baseline, .87 at mid-intervention, .88 at post-intervention and .88 at follow-up.

### Therapists and adherence

Training sessions were delivered by mindfulness teachers, who were also experienced clinicians, either on the national register or who comply with the Good Practice Guidelines. Trainers were given a minimum of two training days with one of the authors (PF), who has extensive experience training professionals to deliver workplace interventions based on ACT. Trainers were supervised by regular group supervisions sessions with one of the authors (CDG), who is an experienced clinician with more than six years of experience.

### Statistical analyses

#### Preliminary analyses

Data analysis was carried out with the statistical package SPSS (version 24) for main analyses and the PROCESS macro for the mediation analyses. Outliers were investigated by using boxplots and histograms. The data was missing completely at random (Χ2 = 932.037, df = 921, p = .39). To replace random missing data simple imputation by using variable mean substitution was used. Intention-to-treat (ITT) analyses with imputation of missing data by last value carried forward approach was adopted for missing follow-up data (e.g. follow-up surveys not completed by participants who dropped out). For completeness, per protocol analyses are also reported. To ascertain whether data met the assumptions for statistical tests for univariate tests, exploratory analyses to assess independence of observations, homogeneity of variance, normality and sphericity were carried out. Multivariate normality, homogeneity of covariance matrices and independence of residuals were tested to assess model fit for multivariate tests. To examine the association between the variables under investigation, correlations were calculated using Pearson’s r coefficient. The correlation coefficients were interpreted as following: r = .10 as a weak effect, r = .25 as a moderate effect and r = .40 as a strong effect size [56].

#### Analyses on primary and secondary outcomes

Several steps were taken to analyse the effect of the intervention on primary and secondary outcomes. Baseline between-group differences were tested by conducting independent sample t-tests between the intervention group and the waitlist control on outcomes and process measures. We performed a mixed repeated measures Analysis of Variance (ANOVA), with Time (baseline, mid-intervention, post intervention and follow-up) as the within-subjects variable and Condition (ACT condition and waitlist control) as the between-subjects factor, to assess whether the level of psychological distress (GHQ-12) decreased in the ACT condition relative to the waitlist control at post-intervention time-points.

A set of multivariate analyses of variances (MANOVAs) with burnout (physical exhaustion, emotional exhaustion and cognitive weariness), worry and rumination (affective rumination and perseverative cognition) and patient safety (perceptions of organisational patient safety and perceptions of personal patient safety) being the dependent variables, were conducted. Pillai’s Trace value was selected given that experimental and control sample sizes were almost equal. When an interaction was significant, univariate tests were explored. Significant interactions were followed-up with tests of simple effects by comparing the means at each time-point with the mean of baseline levels or by comparing the mean of each level to the mean of following levels (baseline vs mid-intervention, mid-intervention vs post-intervention, post-intervention vs follow-up). Follow-up tests of simple effects were chosen over one-way ANOVAs or paired-sample t tests to avoid increasing the probability of Type I errors. When interaction effects were not significant, follow-up tests of the main effects (post hoc tests and pairwise comparisons) were reported. Effect sizes were reported with partial eta squared (η2), that were interpreted as η2 = 0.01 small, η2 = 0.09 medium, and η2 = 0.25 large effect sizes.

#### Mediation analyses

To test the hypothesis that changes in psychological distress at post-intervention were mediated by changes in the ACT processes throughout the intervention, eight bootstrapped mediator models were tested. A bootstrapping approach [57] was preferred given the size of the sample [58], and to test indirect effects [59]. Similarly to Lloyd and colleagues [23], in these models, we tested for the effect of the ACT intervention (predictor vs waitlist control) on changes of psychological distress (baseline to post-intervention) through changes in the ACT processes of mindfulness, values obstruction, values progression, self-compassion (from mid-intervention to post-intervention). A final model was assessed to test whether the ACT intervention had an indirect effect on patient safety perceptions related to work conditions (individual perceptions of organisational patient safety) and to one’s self (individual perceptions of personal patient safety) (baseline to post-intervention) via reductions in psychological distress and burnout (mid-intervention to post-intervention).

#### Reliable and clinically significant changes

We assessed the proportion of participants in intervention and control arms reporting reliable and clinically significant changes in the GHQ via the Leeds Reliable Change Indicator [60] group version. This calculator uses the methods for calculating reliable and clinically significant change delineated by Jacobson and Truax [61]. Here reliable change is considered to have occurred if change is greater than measurement error; whereas, additionally clinically significant change is considered to have occurred if the change also reflects a remission of the problem, based on comparison to the distribution of scores in a ‘normative’ sample without the condition of interest or to an a priori external criterion.

For calculation of the threshold for reliable change, measurement error for the GHQ was based on the Cronbach’s alpha reported in a published validation study [62]. The mean and standard deviation were taken from the same study, representing the mean for detecting depression in the general population (major depression, Mean = 5.3 SD = 4.2).

For calculation of clinically significant change, we additionally included comparison norms from a [63, 64] representative population survey comprising 213, 365 data points (Mean = 1.938; SD = 0.463). The norms of the clinical and normative group do not overlap. Therefore, as suggested by Jacobson and Truax [61], we used Criterion b as the basis for calculating clinically significant change.

## Results

### Preliminary analyses

Descriptive statistics are presented in Table 1 for participants who returned consent and the baseline questionnaire (n = 98). At baseline, the mean score for psychological distress (GHQ-12) was 16.04 (SD = 4.85) which is near that of highly stressed HCPs groups (McConachie et al., 2014). Before the start of the intervention 16.3% of the sample was classified as asymptomatic, 23.5% was sub-clinically symptomatic, 29.6% symptomatic and 30.6% was highly asymptomatic.

**Table 1:** Intention to treat (ITT) means (standard deviations) for outcome and process measures for all participants in the ACT (n = 52) and waitlist control (n = 46) groups.

	Baseline		Mid-intervention		Post-intervention		Follow-up	
	ACT	Waitlist	ACT	Waitlist	ACT	Waitlist	ACT	Waitlist
Psychological distress	16.04 (4.85)	15.48 5.59	11.65 (5.13)	14.63 (5.44)	9.89 (4.90)	13.46 (5.96)	11.15 (7.53)	13.54 (8.97)
Physical fatigue	4.20 (1.25)	4.34 (1.00)	3.53 (1.00)	3.96 (1.03)	3.60 (1.15)	4.10 (1.30)	3.42 (1.17)	3.93 (1.29)
Emotional exhaustion	2.87 (1.22)	2.83 (1.43)	2.87 (1.22)	2.83 (1.44)	2.48 (1.20)	2.73 (1.46)	2.43 (1.36)	2.65 (1.33)
Cognitive weariness	4.05 (1.37)	3.84 (1.28)	3.46 (1.30)	3.92 (1.29)	3.28 (1.18)	3.60 (1.20)	3.22 (1.30)	3.70 (1.15)
Worry and rumination	3.09 (.98)	2.96 (.89)	2.87 (.95)	2.89 (1.00)	2.67 (.92)	2.76 (1.10)	2.64 (0.96)	2.74 (0.97)
Patient safety (organisational)	1.94 (1.21)	2.04 (1.11)	1.89 (1.09)	2.02 (1.22)	1.67 (1.04)	1.87 (1.07)	1.79 (1.07)	1.87 (1.07)
Patient safety (personal)	4.23 (.96)	4.09 (.96)	4.08 (1.23)	4.00 (1.26)	4.15 (1.19)	4.26 (1.04)	4.28 (.95)	4.37 (.88)
Mindfulness	43.52 (7.57)	44.39 (9.69)	44.40 (8.13)	44.11 (9.34)	47.14 (8.70)	44.09 (9.63)	48.65 (7.76)	46.20 (9.90)
Self-compassion	34.40 (4.83)	35.24 (6.33)	33.64 (6.30)	35.04 (7.79)	35.17 (8.39)	33.96 (8.57)	36.52 (7.98)	35.74 (8.61)
Values (obstruction)	19.21 (5.24)	18.78 (6.25)	18.19 (5.72)	19.08 (6.41)	16.23 (5.54)	18.65 (5.97)	16.94 (5.44)	17.30 (5.11)
Values (progress)	20.93 (5.82)	21.17 (6.03)	22.94 (6.00)	21.44 (5.94)	23.23 (5.96)	22.59 (6.05)	23.02 (5.56)	23.86 (5.07)
Distress endurance	41.08 (7.92)	41.24 (7.07)	40.21 (7.81)	40.41 (6.40)	39.90 (8.81)	40.94 (5.67)	41.00 (7.49)	41.05 (6.38)

The data were screened, and no significant outliers were detected. Levene’s test showed that the homogeneity of variances was satisfied. Variables of interest were normally distributed in a manner consistent with normality—skewness and kurtosis levels did not exceed the cut-off values (asymmetry < 2 and kurtosis < 7) provided by Curran, West and Finch [65]. Mauchly’s test indicated that the sphericity assumption for the analysis was not satisfied, χ2 (5) = .55, *p* = < .001, ε = .71 (Greenhouse-Geisser), therefore, we used adjusted univariate significance tests (Greenhouse-Geisser). Table 1 reports the means and standard deviations for each variable. One-way ANOVAs revealed no significant differences between groups on the baseline measurements confirming baseline equivalence. S2 reports the correlations between study measures.

### Primary outcomes: Psychological distress

The 4 x 2 mixed ANOVA indicated a significant interaction between Time and Condition, F (2.12, 203) = 3.49, *p* = .03, the magnitude of which was consistent with a small effect size, partial η2 = .04. There was also a main effect of Time, F (2.12, 203) = 13.27, *p* = < .001, partial η2 = .12, and a significant main effect of the Condition, F (1, 96) = 5.26, *p* = .02, partial η2 = .05. This indicates that the ACT training had a beneficial effect on people’s psychological distress in comparison to controls. To decompose this interaction, tests of simple effects revealed this interaction to be significant from baseline to mid-intervention, F (1, 96) = 8.94, *p* = .004, η2 = 0.9 and from baseline to post-intervention F (1, 96) = 8.96, p = .004, partial η2 = 0.9, but the interaction was not significant from baseline to follow-up F (1, 96) = 1.50, *p* = .22, partial η2 = 0.2.

**Fig 2:**
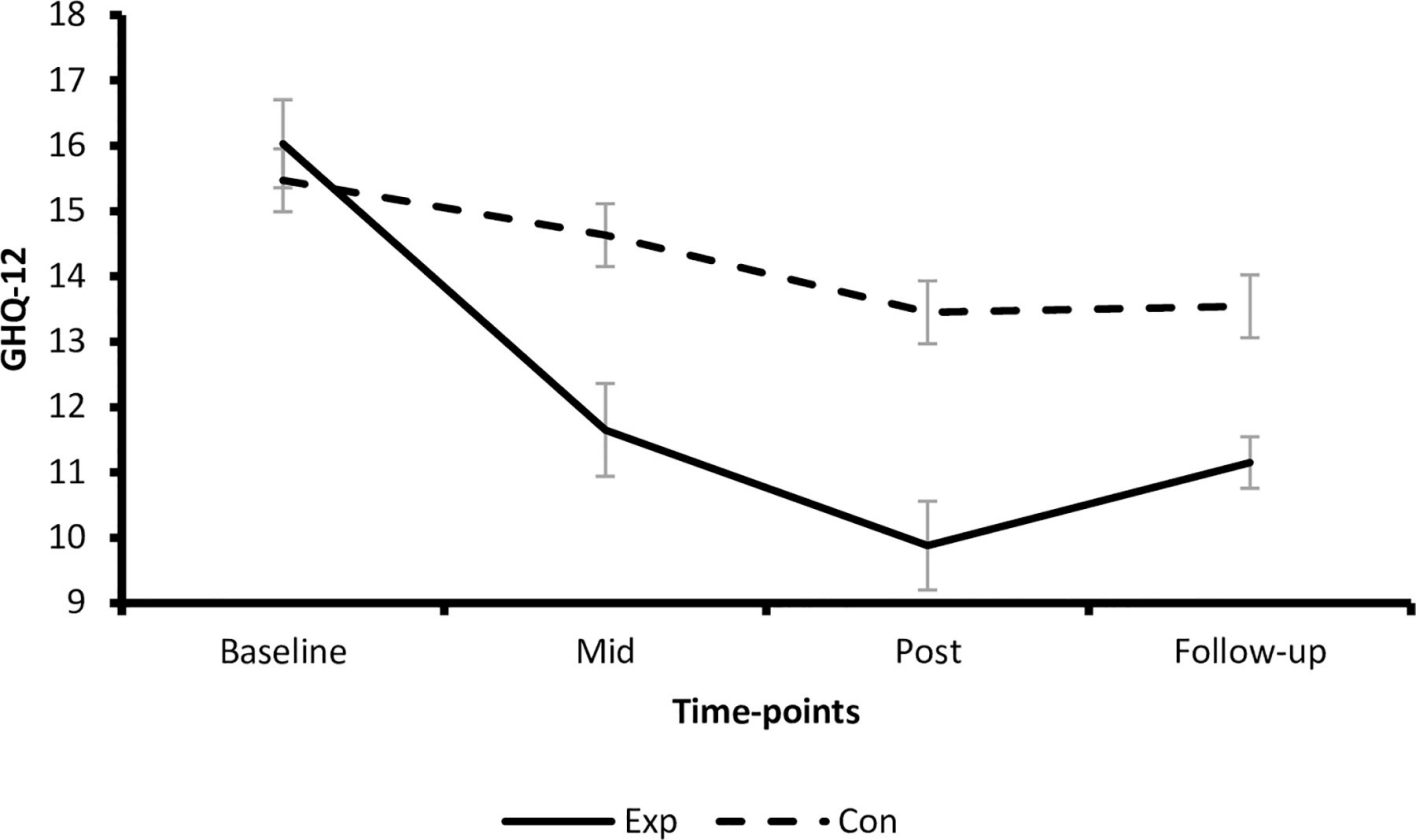
Psychological distress (GHQ-12) scores (and standard errors).

In the ACT group, repeated within-subjects contrasts revealed a change from baseline to mid-intervention, F (1, 51) = 27.38, *p* = < .001, partial η2 = .35, post-intervention, F (1, 51) = 39.59, *p* = .01, partial η2 = .44, and follow-up, F (1, 51) = 350.65, *p* = < .001, partial η2 = .87, indicating that the level of psychological distress decreased across time during the intervention. In the waitlist control, repeated within-subjects contrasts did not reveal a change from baseline to mid-intervention, F (1, 45) = 1.05, *p* = .31, partial η2 = .02, but from baseline to post-intervention, F (1, 45) = 4.39, *p* = .04, partial η2 = .09, and follow-up, F (1, 45) = 225.13, *p* = < .001, partial η2 = .83, indicating that the level of psychological distress decreased across time during the intervention. For a visual representation of the primary outcomes findings see Fig 2.

### Per protocol analyses

The means and standard deviations of the per protocol analyses are presented in Table 2.

**Table 2:** Per protocol analyses.

Condition	Mean	SD
**Experimental**		
Baseline	16.13	4.85
Mid-intervention	10.00	5.75
Post-intervention	9.51	5.62
Follow-up	10.53	7.81
**Control**		
Baseline	15.44	5.65
Mid-intervention	11.33	6.48
Post-intervention	13.71	5.49
Follow-up	12.93	9.57

When the data for all participants were examined, significant group×time interactions were still observed for the GHQ-12 from baseline to post-intervention, F(1, 40) = 4.80, p = .034. However, the groupxtime interaction was no longer observed from baseline to mid-intervention, F(1, 40) = 1.75, p = 0.193 or from baseline to follow-up, F(1, 40) = .452, p = 0.505.

### Secondary outcomes: Burnout

A repeated-measures MANOVA on the burnout sub-components of physical exhaustion, emotional exhaustion, and cognitive weariness found a significant Time x Condition interaction using Greenhouse-Geisser, F (2.32, 223.21) = 3.82, *p* = .02, partial η2 = .04. Univariate analyses showed that cognitive weariness significantly declined from baseline to mid-intervention, F (1, 96) = 8.48, *p* = .004, partial η2 = .08, post-intervention, F (1, 96) = 4.52, *p* = .04, partial η2 = .05, and follow-up, F (1, 96) = 5.49, *p* = .02, partial η2 = .05.

There was also a significant effect of Time, F (2.28; 218.40) = 11.64, *p* = < .001, partial η2 = .11 and Condition, F (1, 96) = 4.31, *p* = .04, partial η2 = .04 for physical exhaustion. For physical exhaustion, the effect of Time was found to be significant from baseline to mid-intervention F (1, 96) = 27.95, *p* = < .001, partial η2 = .23, post-intervention, F (1, 96) = 11.17, *p* = .001, partial η2 = .10, and follow-up, F (1, 96) = 18.35, *p* = < .001, partial η2 = .16. A significant interaction between Time x Condition was not found, F (2.27, 218.40) = 1.23, *p* = .30.

A significant effect for Time was also found for emotional exhaustion F (1.56, 150.02) = 3.91, *p* = .03, partial η2 = .04. This effect was significant from baseline to mid-intervention, F (1, 96) = 27.95, *p* = < .001, partial η2 = .23, and significant from baseline to post-intervention, F (1, 96) = 27.95, *p* = < .001, partial η2 = .23, and follow-up, F (1, 96) = 27.95, *p* = < .001, partial η2 = .23. No significant effect was found for Condition, F (1, 96) = .93, *p* = .34 or Time x Condition, F (1.87, 187.03) = .96, *p* = .37.

### Secondary outcomes: Work-related worry and rumination

Repeated-measures MANOVA on worry and rumination (affective rumination, perseverative cognition) showed a significant main effect of Time, F (3, 288) = 5.26, *p* = .004, partial η2 = .05. However, no significant main effect was found for either Condition, F (1, 96) = .00, *p* = 1.00, or Time x Condition interaction, F (3, 288) = .980, *p* = .420. The analyses indicated that a significant effect of Time was found on worry and rumination from baseline to post-intervention F (1, 96) = 7.85, *p* = .006, partial η2 = .08 and from baseline to follow-up, F (1, 96) = 8.67, *p* = .004, partial η2 = .08 indicating that worry and rumination decreased over time. No effect of Time was found from baseline to mid-intervention, F (1, 96) = 3.12, *p* = .08, partial η2 = .03.

### Secondary outcomes: Perceived patient safety

Repeated-measures MANOVA on patient safety (individual perceptions of organisational and personal patient safety) did not show a significant main effect of Time, F (6, 91) = 2.219, *p* = .099, or Condition, F (1, 96) = .477, *p* = .492 or Time x Condition, F (6, 91) = 1.33, *p* = .910 indicating that there was no difference in the perceptions of patient safety at an organisational or individual level.

### Mediation analyses

Results from the mediation analyses indicated that two of the eight models tested, were statistically significant. First, as shown in Table 3, there was an effect of the ACT intervention on psychological distress via changes in the ACT processes of values obstruction (estimate = .17, BCa 95% CI [.02; .33]), mindfulness (estimate = .20, BCa 95% CI [.04; .37]) and self-compassion (estimate = .13, BCa 95% CI [.01; .31]) from mid-intervention to post-intervention. Secondly, there was an effect of the intervention on cognitive weariness via changes in the ACT processes of mindfulness (estimate = .22, BCa 95% CI [.04; .40]), values obstruction (estimate = .14, BCa 95% CI [.01; .30]) and self-compassion (estimate = .17, BCa 95% CI [.01; .37]) from mid-intervention to post-intervention. Lastly, an indirect effect of the intervention was found on patient safety perceptions (baseline to post-intervention) (estimate = -.09, BCa 95% CI [.01; .22]) through a decrease in psychological distress (mid-intervention to post-intervention).

**Table 3:** Bootstrapped simple mediation models testing the indirect effect of the intervention on mental health outcomes (psychological distress and burnout) and patient safety.

Outcome variable	Mediator variable	Bootstrap estimate		BCa 95% CI	
		Estimate	SE	Lower	Upper
**Psychological distress**	**Mindfulness**				
T1-T3	T2-T3	**.20**	**.08**	**.04**	**.37**
Psychological distress	Values progression				
T1-T3	T2-T3	-.03	.08	-.21	.12
**Psychological distress**	**Values obstruction**				
T1-T3	T2-T3	**.17**	**.07**	**.02**	**.33**
**Psychological distress**	**Self-Compassion**				
**T1-T3**	**T2-T3**	**.13**	**.08**	**.01**	**.31**
Psychological distress	Experiential avoidance				
T1-T3	T2-T3	-.01	.04	-.09	.10
**Cognitive weariness**	**Mindfulness**				
T1-T3	T2-T3	**.22**	**.09**	**.04**	**.40**
Cognitive weariness	Values progression				
T1-T3	T2-T3	-.03	.06	-.16	.08
**Cognitive weariness**	**Values obstruction**				
T1-T3	T2-T3	**.14**	**.07**	**.01**	**.30**
**Cognitive weariness**	**Self-Compassion**				
**T1-T3**	**T2-T3**	**.17**	**.09**	**.01**	**.37**
Cognitive weariness	Experiential avoidance				
T1-T3	T2-T3	-.00	.03	-.06	.08
**Patient Safety (organis)**	**Psychological distress**				
T1-T3	T2-T3	**.09**	**.05**	**.01**	**.22**
Patient Safety (personal)	Psychological distress				
T1-T3	T2-T3	-.06	.05	-.19	.00
Patient Safety (organisat)	Cognitive weariness				
T1-T3	T2-T3	.00	.05	-.09	.14
Patient Safety (personal)	Cognitive weariness				
T1-T3	T2-T3	-.00	.05	-.12	.09

Note. BCa = biased corrected confidence intervals. Partially standardised estimates are presented.

### Reliable and clinically significant changes

At four-week post-intervention, 48% (25 of 52) participants who received the ACT intervention met the criteria for "reliable change" with all but one of these changes reaching the threshold for clinical significance 24/52 (46.15%); while, 42% (22 of 52) did not change, and 10% (5 of 52) reliably deteriorated. Spontaneous improvements were also found in the control group: 21.74% (10 of 46) showed reliable improvement, with 19.57% (9 out of 46) reaching the threshold for clinical significance. 10.87% deteriorated (5 of 46).

At follow-up, 52% (27 of 52) of the participants who received the ACT intervention met the criteria for "reliable change” with all but one of these changes reaching the threshold for clinical significance 26/52 (50%), while, 40% (21 of 52) did not change, and 7% (4 of 52) reliably deteriorated. Spontaneous improvements were also found in the control group at follow-up: 34.78% (16 of 46) showed reliable improvement, with 30.43% (14 out of 46) reaching the threshold for clinical significance. 8.70% deteriorated (4 of 46).

## Discussion

This study aimed to test the effectiveness of an ACT-based workplace intervention for improving psychological distress (primary outcome) and burnout, work-related worry and rumination and perceived patient safety (secondary outcomes) in healthcare staff. The results showed that the primary outcome, psychological distress, showed a significant improvement in the ACT treatment arm compared to a waitlist control. However, for the secondary outcomes, the ACT intervention only led to significant improvements in the cognitive weariness aspect of burnout. No statistically significant main effects of the ACT intervention were found for work-related worry and rumination, perceived safety practices or for the physical or emotional exhaustion components of burnout. In support of hypothesis 2, mindfulness, values and self-compassion mediated improvements in psychological distress and the cognitive weariness aspect of burnout. The impact of the ACT intervention was found to be moderated by initial levels of distress with those with high levels of psychological distress tending to respond better to the intervention. Lastly, reliable and clinically significant changes in psychological distress were apparent in larger proportions of those in the ACT arm, with twice as many showing reliable change in the intervention group compared to controls and very few deteriorations.

Overall, our expectations for the first hypothesis were partially met. As predicted, we found a significant condition by time interaction for the primary outcome, psychological distress. This finding is consistent with the positive findings of ACT interventions for distress observed in a number of RCT studies in workplace contexts [12, 15, 24, 29, 66, 67]. Our finding is important because the intervention was conducted with NHS staff, who are at particular risk of experiencing poor wellbeing and burnout [4]. The effect size from the primary outcome compares well with a recent meta-analysis of ACT interventions for improving distress and work-related distress in HCPs [68]. This was the first systematic review and meta-analysis investigating general distress and work-related distress in HCPs, including medical and non-medical professionals. Twenty-two studies met the inclusion criteria in the systematic review, ten studies were included in the meta-analysis. This meta-analysis found that overall ACT interventions outperformed control conditions with a small effect size (g = .39, CIs [.040; .748]) [68]. This study also found that ACT interventions were effective for reducing work-related stress at follow-up. The current study compares well with a cross-sectional study conducted to explore the predictive influences of psychological flexibility (mindfulness and values) and self-compassion on psychological distress, burnout and perceived patient practices [69]. This study suggested that mindfulness-based programmes delivered in the workplace could be enhanced by including a values-based behavioural activation component and that ACT programmes for healthcare staff could benefit from the explicit inclusion of strategies designed to cultivate self-compassion.

For the secondary outcomes, there was evidence of a small effect for burnout. This finding is consistent with a few ACT studies in the workplace [23, 27, 67]. However, our study is the first that aimed to investigate burnout by employing the Shirom Melamed Burnout Measure–which assessed a key component of burnout, cognitive weariness. Our finding suggests that burnout is likely to be related to psychological cognitive functioning, and therefore, is more amendable to change following an ACT intervention. In contrast, for the emotional exhaustion component of burnout, the lack of between-group differences may be because the ACT intervention does not target organisational factors that are likely to contribute to the development of emotional exhaustion. This is consistent with the Conservation of Resources theory that conceptualises emotional exhaustion as related to work, with a focus on the context and organisational settings. Indeed, emotional exhaustion is often the result of chronic exposure to a stressful and demanding work environment, and therefore, will be more difficult to change at an individual level [70].

Given the positive change in the primary outcome, it was surprising that the ACT intervention did not yield benefits for the burnout-related component of work-related worry and rumination. The lack of significance of work-related worry and rumination may depend on the nature of the intervention delivered which specifically aimed to improve psychological flexibility, and not worry and rumination. Although a bulk of research has shown that the ACT training has potential to decrease worry and rumination [71], the interventions delivered in these studies aimed to specifically improve repetitive negative thinking. In contrast, the intervention delivered in this trial aimed to specifically improve mindfulness, values and cognitive defusion skills. Cognitive defusion is specifically designed to help people disentangle from the content of troubling or currently unhelpful thoughts and cultivate the capacity to notice such thoughts from a decentred perspective as passing events in the mind [20]. Although it was not assessed, it may be that cognitive defusion skills, or the believability of negative thoughts, may have improved, as shown in several ACT interventions in the workplace [16, 27, 66, 72–74].

This study did not show a direct effect of the ACT intervention on patient safety. However, for the first time, this study showed that the ACT intervention led to a significant reduction in perceived patient safety via reduction of psychological distress. This finding is in line with the patient safety literature [2, 4, 6] that suggests that psychological distress is a strong proxy of perceived safety practices. Although this finding needs to be replicated in a larger trial, this result is important and suggests that ACT training programmes may have an indirect influence on patients. It may be indeed that skills HCPs learn during the training programmes are transferrable and utilised in clinical practice.

The significant mediation effects of mindfulness and values are consistent with ACT’s underlying theory [11] suggesting that the intervention works through improvements in mindful awareness and valued living. These results are also consistent with previous ACT interventions in the workplace [12, 29, 75]. Also, this result has direct implications for third-wave behavioural therapies more generally (e.g., mindfulness based cognitive therapy or mindfulness-based stress reduction) providing evidence that interventions that include mindfulness-based exercises in their protocol may be effective for NHS staff. It is worth noting how our study differs from previous ACT interventions in the workplace. Although previous studies have found an indirect effect of mindfulness [29] or psychological flexibility [12, 75], to our knowledge, this is the first study to show a significant improvement in psychological distress of NHS staff through an improvement of values (specifically there was an influence on values obstruction facet). This finding suggests that ACT programmes for NHS staff would benefit from integrating mindfulness and values components. This study also found that an ACT-based intervention improved psychological distress and burnout via self-compassion. This finding suggests that ACT-workplace interventions with the inclusion of self-compassion techniques may be beneficial for targeting these outcomes.

Investigation in this RCT of reliable and clinically significant change on the GHQ confirms preliminary findings from previous studies in workplace contexts. In the present study reliable and clinically significant changes occurred for a large proportion of participants (46%), with twice as many participants showing reliable change in psychological distress following the intervention than spontaneous improvements evident in the waitlist control. Similarly, Flaxman and Bond [19] found that 69% of the distressed employees who received the ACT programme improved with a clinically significant change. Brinkborg et al. [24] found that 42% of social workers who received the ACT training improved with a clinically significant change at post-intervention. This finding is important and confirms that ACT training programmes may be particularly helpful for employees who are experiencing psychological distress. The results from our trial suggests that future research should investigate whether this intervention may be particularly helpful for those groups with mental health conditions (e.g., employees with affective disorders disorders). Also, few deteriorations and the same number of participants in the control group suggests that the ACT intervention is unlikely to be harmful.

We recognise there are a number of shortcomings of this current study. First, the level of attrition of participants was relatively high. Of the 98 participants who completed the baseline survey, 63 completed four time-points. Although it is possible to hypothesise that NHS staff who did not find the intervention beneficial were less disposed to continue, there were no significant differences between participants who only completed baseline and those who completed baseline and at least another survey. Also, to limit the impact of the drop-outs, in the current study we employed intention-to-treat analyses with last observation carried forward. Although this is a simple method to account for missing data in repeated-measures studies that maximizes the number of observations, it can inflate the probability of finding a significant intervention effect when it does not exist; therefore we urge some caution until the current findings are replicated in a future, larger scale investigation [76]. That said, as noted earlier in the results, the main findings of the study were substantively the same when using per protocol analyses. Missing data in a RCT may be a threat, therefore, future research ought to explore further the barriers that NHS staff may encounter when undertaking ACT training (e.g., workload, high turnover, job sickness, schedule, and reminders for questionnaire completion). With regards to patient safety practices, it is possible that the heterogeneous nature of the sample with 20.7% participants working in a managerial or administration roles and without direct contact with patients may have diluted or impacted this outcome. Also, we are aware of the limitations of including a single-item measure of perceived patient safety and that including a more objective measure would have been desirable. Lastly, the current study included a waitlist control group as a comparator condition which can be considered an “inactive” control—thus not as strong a comparator when compared to an active control arm. As suggested in a recent review of ACT interventions for improving mental health in healthcare professionals [68], it is more likely to find higher effect sizes in mental health outcomes when ACT interventions are compared to inactive control groups (e.g. other validated interventions) rather than active control groups.

The findings from this RCT show that an acceptance-based approach like ACT may be useful for stressed HCPs, and that self-compassion should be integrated into interventions aimed at reducing staff burnout. Given the effectiveness of the ACT framework, primary care trusts should consider making the intervention available to all staff, and research funders should prioritise HCPs wellbeing. Improving HCPs’ health and wellbeing with ACT training programmes may ultimately result in financial savings for the NHS, through reduced absenteeism rates.

Future research should consider the inclusion of a larger sample, aim to incorporate objective measures of patient safety outcomes and may want to consider the inclusion of self-compassion in the ACT intervention protocol and to investigate, in a larger trial, which aspects of self-compassion (self-kindness, common humanity, mindfulness) change as a result of the intervention. Moreover, researchers ought to consider developing and testing the feasibility of online, digital versions of the current ACT intervention in order to increase accessibility and reach and to ensure easy roll out in any future pandemics [77].

## Conclusion

This study showed that a 4-session ACT training programme is an effective intervention for improving psychological distress in HCPs working within the NHS, including GPs, nurses, and managers. Mindfulness, values and self-compassion were found to mediate intervention effectiveness. Reliable and clinically significant changes were found in 46% of the participants receiving the intervention. These findings should now be confirmed in a larger scale randomised controlled trial.

## Supporting information

S1 Checklist (DOC)

S1 File Content of the ACT intervention programme. (DOCX)

S2 File Pearson’s product moment correlations between study measures. (TIF)

S1 Protocol (DOCX)
