# Supplementary material for: A workplace Acceptance and Commitment Therapy (ACT) intervention for improving healthcare staff psychological distress: A randomised controlled trial
Source: PLoS One. 2022 Apr 20;17(4):e0266357. doi: 10.1371/journal.pone.0266357 (PMC9020690; doi:10.1371/journal.pone.0266357)
Supplement: S1 File — (DOCX) [file pone.0266357.s002.docx]

**S1 File. Content of the ACT intervention programme.**

| **SESSION 1** | **SESSION 2** | **SESSION 3** | **SESSION 4** |
| --- | --- | --- | --- |
| **Welcome** | **Welcome back** | **Welcome back** | **Welcome back** |
| Aim of the training  (three column theoretical framework) | Aim of the training  (three column theoretical framework) | Aim of the training  (three column theoretical framework) | Aim of the training  (three column theoretical framework) |
| **Introduction to mindfulness** | **Mindfulness practice** | **Mindfulness practice** | **Mindfulness practice** |
| Raisin exercise | Body and breath exercise (difference between “sensing” and “thinking” modes of Mind) | Body and breath exercise  (focus on reconnecting with body) | Body and breath exercise  (waking up from the autopilot, difference between “sensing” and “thinking” modes of Mind, focus on reconnecting with body) |
| Mindfulness psychoeducation | **Homework review** | **Homework revision** | **Homework review** |
| How to practice mindfulness | Review of mindfulness practice and value-based action practice | Review of mindfulness practice and value-based action practice | Review of mindfulness practice and value-based action practice |
| Body and breath exercise (waking up from the autopilot) | **Cultivating psychological flexibility** | Reflection on the ACT matrix exercise | Reflection on the ACT matrix exercise |
| **Introduction to value-based actions** | ACT matrix | **Cultivating acceptance** | **Cultivating values** |
| Values psychoeducation | **Cultivating values** | The physicalizing exercise | Different version of the ACT matrix (personally chosen values) |
| Values card sort exercise | Different version of the values card sort exercise | Rumi’s guest house poem | **Cultivating psychological flexibility** |
| **How to overcome issues with practice** | Identification of value-based actions | **Cultivating values** | Passengers on the bus exercise in group |
| Normalisation | **Cultivating cognitive defusion** | Different version of the ACT matrix (health, relationships, work and career, leisure time) | **Training programme review** |
| Passengers on the bus exercise | Defusion psychoeducation | **Homework** | Aim of the training  (three column theoretical framework)  ACT Matrix  What to take away  How to keep practicing |
| **Homework** | Normalisation | Encouragement to practice mindfulness 5 times a week | **Conclusion** |
| Daily mindful activity | Three-step exercise | Encouragement to reflect on personal values | Poem (The Coconut by Paul Hostovsky) |
| Encouragement to practice mindfulness 5 times a week | **Homework** | Noticing “towards” and “away” moves in daily life |  |
| Start noticing “towards” moves | Daily mindful activity | **Conclusion** |  |
| **Conclusion** | Encouragement to practice mindfulness 5 times a week | Three step mindfulness exercise |  |
| Two sheets of paper exercise (importance on values) | Start noticing a “towards” and an “away” move |  |  |
|  | **Conclusion** |  |  |
|  | Two sheets of paper exercise  (importance on coexistence of values and unhelpful thoughts) |  |  |
