# Supplementary figures and images for: A workplace Acceptance and Commitment Therapy (ACT) intervention for improving healthcare staff psychological distress: A randomised controlled trial
Source: PLoS One. 2022 Apr 20;17(4):e0266357. doi: 10.1371/journal.pone.0266357 (PMC9020690; doi:10.1371/journal.pone.0266357)

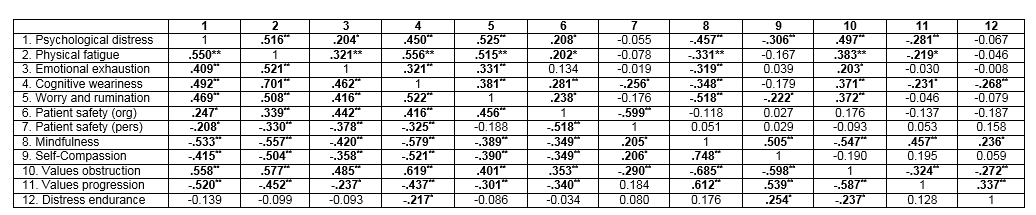

Supplement: S2 File — (TIF) [file pone.0266357.s003.tif]
