# Supplementary material for: A workplace Acceptance and Commitment Therapy (ACT) intervention for improving healthcare staff psychological distress: A randomised controlled trial
Source: PLoS One. 2022 Apr 20;17(4):e0266357. doi: 10.1371/journal.pone.0266357 (PMC9020690; doi:10.1371/journal.pone.0266357)
Supplement: S1 Protocol — (DOCX) [file pone.0266357.s004.docx]

**Protocol: An Acceptance and Commitment Therapy-based workplace intervention for improving the well-being of NHS primary care staff**

**(IRAS Project ID 228214)**

*Background*

Workforce surveys in the United Kingdom suggest that between 25% and 40% of workers in various occupations would meet the diagnostic criteria for a minor psychiatric disorder (Hardy, Woods, & Wall, 2003; Stride, Wall, & Catley, 2007; Wall et al., 1997). In terms of cost, higher levels of employee distress have been associated with an elevation in

both sickness absence and work cutback days, where distressed employees are present at work but unable to perform effectively (Hardy et al., 2003; Kessler & Frank, 1997; Kessler, Merikangas, & Wang, 2008). In view of these observations, it is concerning that only a small fraction of distressed employees receive evidence -based interventions in or out of the workplace (Hilton et al., 2008; White, 2000). For example, Hilton et al. (2008) recently estimated that 78% of Australian workers with clinically significant distress were not currently receiving g any recognised support, and the picture seems to be similar in the UK workforce (White, 2000). Indeed changes in the organisation and the management of health care provision in the UK, coupled with the nature of medical practice have increased the experience of stress, burnout and poor psychological wellbeing in general practitioners and health professionals more generally (Gibson et al. 2015). Moreover, these high levels of distress are likely to have resultant implications for patient care (e.g., reduced patient safety and increased medical error) and for the health professionals’ health and wellbeing (Hall et al., 2016; O’Connor et al., 2000). Taken together, these findings underscore the importance of implementing and evaluating interventions that have the potential to improve mental health in the workplace, particularly within the NHS.

In order to prevent and reduce distress among employees, researchers have begun implementing and evaluating mindfulness-based worksite training interventions. One of these interventions is based on Acceptance and Commitment Therapy (ACT; Hayes et al., 1999). ACT is a mindfulness-based intervention that seeks to change the way people respond to stress- related thoughts and feelings. Unlike traditional stress management approaches (such as relaxation training), ACT does not seek to reduce employees’ unwanted thoughts and emotions. Instead, ACT increases psychological flexibility, which refers to the ability to pursue valued work and life goals even when experiencing difficult thoughts and emotions.

Previous pilot studies have suggested that several (at least three) half day sessions of training in ACT principles and techniques can lead to clinically meaningful reductions in employee distress and increases in levels of psychological flexibility. ACT interventions also appear to affect improvements in various other indicators of employee well-being, such as the ability to be innovative at work (Bond & Bunce, 2000; Dahl et al., 2004; Flaxman & Bond, 2006; 2010a; 2010b; Varra et al., 2008). Moreover, higher levels of psychological flexibility have been associated not only with improved mental health, but also with improved job performance and absenteeism in UK workers (Bond & Bunce, 2003; Bond, Flaxman, & Bunce, 2008).

To ascertain whether these beneficial effects of ACT might also apply to NHS primary care staff, this project will implement and evaluate an ACT -based worksite intervention for improving well-being, work-related mental health and patient safety in this group. The programs will be implemented in a brief form across four consecutive two hour sessions. This will be the first study evaluating the efficacy of an ACT-based workplace intervention in NHS primary care settings. Based on previous research, it is predicted that ACT will lead to significant increases in general well - being, work related mental health and psychological flexibility. In this study we will also assess whether there is a perceived positive influence on clinical practice, via improvements in safe clinical practice.

*Hypotheses*

1) Compared to a wait-list control group, those receiving ACT will have significantly better scores on primary and secondary outcome measures at post-intervention time-points.

2) Improvements in outcomes will be mediated by improvements in psychological flexibility.

**METHODS**

*Design*

The study will employ a randomised controlled design to examine the effectiveness of a group-based ACT training programme for improving the well-being of NHS staff, compared to a wait-list control group.

*Participants*

Participants will be primary care staff within the NHS in Leeds and West Yorkshire. There will be one inclusion criterion: currently employed by the NHS and working within NHS primary care settings in Yorkshire and Humber. There will also be one exclusion criterion: not presently at work.

Based on sample sizes from other ACT studies in similar settings (e.g., Flaxman & Bond, 2010; Flaxman et al., 2016) we aim to recruit 140 people.

*Procedure*

Recruitment: The study will be advertised to potential participants via local awareness raising in primary care settings. Participants will be given information about the study and will be given the e-mail address of a member of the research team (NHS e-mail) to contact a member of the research team if interested in opting-in to the study.

Once participants have expressed an interest in the study, they will immediately be sent the participant information sheet (PIS) via e-mail, which outlines the nature of the research and training. After having had at least 24 hours to consider the information presented in the PIS, consent will be taken online. A consent form will be the first sheet of the pre-intervention questionnaire. Immediately after giving consent participants will then enter demographic details and generate their own individual participant number (First two letters of their mother's maiden name initials and the last four digits of their telephone number). This reference number will be used to complete all online questionnaire batteries.

Participants will be informed that they will shortly be randomly allocated to begin the training, the first session beginning in late September 2017, or to the waitlist control group. As soon as an initial 40 people have volunteered, the first randomisation process will take place, allocating volunteers to the ACT training programme or to the waiting list control group. Participants allocated to the training group will then receive dates, times, and locations of the scheduled training sessions. Participants will be randomised to groups by the research team using a computer generated randomisation procedure.

Those allocated to the waiting list control will begin the training in Feb /March 2018. These participants will receive an email (or letter) providing reasons for being allocated to the later training group (i.e., limitations on the number of people that can be trained at one time, and the need to rigorously evaluate the impact of the training on staff well - being). All participants will be required to complete an online questionnaire battery (described in more detail below) on four separate occasions over the 14 week evaluation period. They will be e-mailed the questionnaire at the appropriate time-point, if they don’t complete the questionnaire within three days, they will be sent a reminder e-mail.

*Intervention*

The intervention will be based on an existing standardised ACT protocol designed for workplace settings. Participants will be invited to attend a total of four two-hour group sessions; these sessions will occur on four consecutive weeks.

Each session will consist of between 8 and 15 people. Training sessions will be delivered by experienced clinicians, who are also trained mindfulness teachers either on the national register or who comply with the Good Practice Guidelines. These trainers will be taught to deliver the study’s ACT intervention over a minimum of two training days with Dr. Paul Flaxman, who has extensive experience training professionals to deliver workplace interventions based around ACT. They will be supervised by Dr Flaxman and by a professional mindfulness supervisor.

*Measurement*

A questionnaire battery will be administered to participants on four occasions:

1. Pre-intervention: minus 1 to 2 weeks

2. Mid-intervention: between sessions 2 and 3 of the intervention (week 3)

3. Post-intervention: one week after session 4 (week 5)

4. Follow-up: 3 months after post-intervention (week 19)

The questionnaire battery will take approximately 25 minutes to complete on each occasion. These on line questionnaires will be completed via encrypted survey software (known as Bristol Online Surveys) hosted by the CI’s institution (University of Leeds). Each participant will receive an email containing a link to the questionnaires. Those allocated to begin the training in in September 2017 will be e-mailed the questionnaires to complete prior to training.

*Primary outcome*

1. General Health Questionnaire 12 (Goldberg & Williams, 1988). The GHQ-12 is one of the most widely used and well-validated measures of general well-being/mental health among working populations. The scale consists of a mix of positively and negatively worded items designed to capture various aspects of a person’s recent psychological functioning (e.g., sleep loss due to worry, ability to play a useful part in things, feeling able to face up to problems).

Higher scores indicate a higher level of psychological distress. As well as providing a primary overall measure of general mental health, the GHQ-12 can also be used to indicate the proportion of the sample that is experiencing clinically relevant level of distress.

*Secondary outcomes*

2. Shirom-Melamed Burnout Measure (SMBM; Shirom, 2003; Shirom & Melamed, 2006). The SMBM will be used to measure job-related burnout. This well-established 14-item scale has three subscales that are designed to capture core components of the burnout syndrome: physical fatigue, emotional exhaustion, and cognitive weariness. We selected this particular measure for the following reasons: (1) its theoretical underpinnings are clearly specified

(Shirom, 2003); (2) it explicitly seeks to capture a construct that is distinct from depression and anxiety; and (3) unlike more generic burnout measures, responses to the SMBM are temporally anchored to the past 30 work days.

3. Work-related worry and rumination during non-work time. Maladaptive perseverative cognition will be measured with the affective rumination subscale of the work-related rumination questionnaire (WRRQ; Cropley, Michalianou, Pravettoni, & Millward, 2012). This 5-item scale captures the degree to which a person is troubled by intrusive work-related thoughts during their leisure time. This subscale has demonstrated good psychometric properties among various samples of UK workers (Cropley et al., 2012; Querstret & Cropley, 2012). This measure will be supplemented with a 5-item work related worry and rumination scale developed and validated by Flaxman and colleagues (Flaxman, 2014; Flaxman et al., 2012). Participants will be asked to report amount of work-related worry/ rumination experienced during non-work time (e.g., evenings and weekends) over the past week.

4. Perceptions of patient safety will be measured using the safe practitioner measure using the following two items “In the past four weeks, my practice is not as safe as it could be because of work related factors/conditions” and “My practice is safe”. This measure has been shown to bereliable and valid (Louch et al., in press; Louch et al., 2016).

*Process of change measures*

1. Multidimensional Experiential Avoidance Questionnaire (MEAQ) – Distress Endurance subscale (Gamez et al., 2011). The 11-item MEAQ-DE subscale measures an important aspect of psychological flexibility: one’s ability to endure difficult thoughts and feelings in order to complete important activities.

2. 15-item Five Facet Mindfulness Questionnaire (Gu et al., 2016; Baer et al., 2006, 2008). This is the recent 15-item reduction (Gu et al., 2016) of the popular 39-item scale (Baer et al., 2006, 2008), which is the most widely used multidimensional measure of mindfulness skills. The FFMQ has 5 subscales: observing (e.g., “When I’m walking, I deliberately notice the sensations of my body moving”); describing (labelling with words; e.g., “I’m good at finding the words to describe my feelings”); acting with awareness (e.g., “I find it difficult to stay focused on what’s happening in the present”); nonjudging of experience (e.g., “I make judgments about whether my thoughts are good or bad”); and nonreactivity to difficult inner experience (e.g., “When I have distressing thoughts or images, I am able just to notice them without reacting”).

3. Valuing Questionnaire (VQ; Smout et al. 2014). The 10-item valuing questionnaire is designed to assess people’s ability to engage in action that is consistent with their personal values, and also their experiences of obstacles to pursuing such actions. The scale has previously demonstrated good psychometric properties in both clinical and nonclinical adult populations.

4. Self-Compassion Scale – short-form (SCS-SF; Raes et al., 2011). This 12-item self-compassion scale has 6 subscales: self-kindness; self-judgement; common humanity; isolation; mindfulness; and over-identification. The measure captures the propensity to treat oneself with care and kindness, to accept one’s imperfections, and tendency

to take a balanced perspective on one’s experiences. We included this scale due to the growing interest in self-compassion as a potential mechanism of change in ACT (and other mindfulness-based) interventions, and the relevance of self/other compassion for professionals (such as GPs) working in healthcare settings (e.g., Cummings, 2014).

*Other measures*

Demographics questionnaire (age, gender, occupation, years in occupation, previous/current mindfulness practice).

*Analyses*

Hypothesis 1: Compared to a wait-list control group, those receiving ACT will have significantly better scores on outcome measures at all post-intervention time-points.

Here we will compare group scores between the control and ACT conditions at all time points using ANOVA. All available data will be included in the analyses, regardless of number of treatment sessions attended. This will involve an intention-to-treat (ITT) approach, with imputation of missing data by last value carried forward.

Hypothesis 2: Improvements in outcomes will be mediated by improvements in psychological flexibility in the ACT condition but not in the wait-list control condition. Here we will use mediation and moderation analysis to test whether change in the psychological flexibility processes helps to explain any changes in outcome variables. We will run this analysis to test if these effects are specific to the intervention group.

**Main Ethical Issues**

*Confidentiality*

The participants will be employees of the NHS primary care organisations in which this research is occurring. They will rate questionnaires capturing information about their well-being, and mood in relation to their working lives. Consequently, it is of paramount importance that the information they give within the questionnaires is confidential. Thus, upon entry to the study - after giving consent - participants will be generate a reference number for themselves (first two letters of mother's maiden name and last four digits of mobile number). This will allow us to separate data given within questionnaires from participant’s contact details and demographics. Once they have given consent their contact details and demographics will be kept on one database, alongside their reference number. A separate database will contain the questionnaire data linked to the reference number alone. This reference number will be used on all data captured in online surveys. They will not enter any personally identifiable information alongside questionnaire data (name, address etc.). In addition, online survey data will be collected via an encrypted website (Bristol Online Surveys). There will also be the opportunity to complete hard-copies of the questionnaires before or after the intervention. Here questionnaires will be completed at the location of the training sessions. Hard-copies of the questionnaire will hold no person identifiable information; they will require only the anonymised participant information number alongside questionnaire responses. Mindfulness practitioners will return hard-copies of the questionnaires to the research team at the University of Leeds, these questionnaires will be stored in secure filing cabinets in locked offices within the university.

Only the research team (O’Connor, Graham and Prudenzi) will have access to person identifiable data. This will be safely stored on a University-issued password protected computer. In addition, to maintain confidentiality amongst participants, the requirement of confidentiality during the training sessions will be emphasized at the beginning of each session. Participants will also be informed that they do not have to share personal information during the training sessions. There will be flexibility in allocating participants to alternative training sessions if they request not to attend with a close colleague or manager. A random selection of training sessions will be recorded to evaluate trainer adherence to the intervention protocols. In this case, participants will be asked for their consent at the beginning of the session. Recordings of training sessions will not be transported, will be uploaded immediately to a university password protected computer via the "desktop anywhere" programme - as accessed by a university laptop. After uploading the session will be erased from the recording device. Recordings of training sessions will also be stored securely on a University computer.

Note, in line with open science initiatives we will, after an embargo period of three years, store the anonymised data within the University of Leeds research data repository.

*Managing Risk*

Based on previous experience of similar intervention research in the workplace (e.g., Flaxman & Bond, 2010), the risk of harm to participants is minimal. No problems have been reported during several similar intervention studies conducted by Dr Flaxman over the past 10 years (including recent studies conducted in other NHS trusts). As stated, past experience, and the focus of the training on occupational functioning as opposed to childhood traumas and wider mental health problems suggests that the disclosure of significant risks (e.g. immediate plans for suicide) or safeguarding issues (e.g risk of harm to vulnerable others) is unlikely. Nonetheless, we cannot rule-out the occurrence of such an event; therefore, we have an agreed escalation protocol (included). This escalation protocol has been approved by local Medical and Nursing Directors in line with ethical guidance for health practitioners relating to risks to their own health and that of other people. Participants and trainers will be asked to

agree to the escalation protocol prior to the intervention.

**References**

Baer, R. A., Smith, G. T., Hopkins, J., Krietemeyer, J., & Toney, L. (2006). Using self-report assessment methods to explore facets of mindfulness. *Assessment*, 13, 27-45. doi: 10.1177/1073191105283504

Baer, R. A., Smith, G. T., Lykins, E., Button, D., Krietemeyer, J., & Sauer, S. (2008). Construct validity of the five facet mindfulness questionnaire in meditating and non-meditating samples. *Assessment*, 15, 329-342. doi: 10.1177/1073191107313003

Cropley, M., Michalianou, G. Pravettoni, G,. & Millward, L. (2012). The relation of post work ruminative thinking with eating behaviour. *Stress and Health, 28*, 23-30.

Cummings. J. (2014). *Putting compassion at the heart of healthcare*. NHS England blog. Retrieved on 11the June 2017 from <https://www.england.nhs.uk/blog/jane-cummings-4/>

Gibson J, Checkland K, Coleman A, Hann M, McCall R, Spooner S, et al. Eight national GP worklife survey. 2015.

Gu, J., Strauss, C., Crane, C., Barnhofer, T., Karl, A., Cavanagh, K., & Kuyken, W. (2016). Examining the factor structure of the 39-item and 15-item versions of the Five Facet Mindfulness Questionnaire before and after mindfulness-based cognitive therapy for people with recurrent depression. *Psychol Assess, 28*(7), 791-802. doi: 10.1037/pas0000263

Hall, L., Johnson, J., Watt, I., Tsipa, A., O’Connor, D.B. (2016). Healthcare staff wellbeing, burnout, and patient safety: A systematic review. *PLoS ONE 11(7):* e0159015. doi:10.1371/journal.pone.0159015.

Flaxman, P. E. (2014). A research journey into perfectionism, perseverative cognition, and acceptance-based therapy. *Keynote address at the British Psychological Society (BPS) Division of Occupational Psychology Annual Conference (Brighton, January, 2014).*

Flaxman, P. E., & Bond, F. W. (2010). Worksite stress management training: Moderated effects and clinical significance. *Journal of Occupational Health Psychology, 15,* 347-358.

Flaxman, P. E**.,** Menard, J., Bond, F. W., & Kinman, G. (2012). Academics’ experiences of a respite from work: Effects of self-critical perfectionism and perseverative cognition on post-respite well-being. *Journal of Applied Psychology, 97,* 854-865*.*

Kenny, D. A., Kashy, D. A., & Bolger, N. 1998. Data analysis in social psychology. In D. T. Gilbert, S. T. Fiske, & G. Lindzey (Eds.), The handbook of social psychology (pp. 233–265). Boston, MA: McGraw-Hill.

Louch, G, O’Hara, J., Gardner, P.H., & O’Connor, D.B. (in press). A daily diary approach to the examination of chronic stress, daily hassles and safety perceptions in hospital nursing. *International Journal of Behavioral Medicine*.

Louch, G, O’Hara, J., Gardner, P.H., & O’Connor, D.B. (2016). The daily relationships between staffing, safety perceptions and personality in hospital nursing: A longitudinal on-line diary study. *International Journal of Nursing Studies*, 59, 27-37.

O’Connor, D.B., O'Connor, R.C., White, B.L. & Bundred, P.E. (2000). The effect of job strain on British general practitioners’ mental health. *Journal of Mental Health*, 9, 637-654

Querstret, D. & Cropley, M. (2012). Exploring the relationship between work-related rumination, sleep quality and work-related fatigue. *Journal of Occupational Health Psychology, 17*, 341-53.

Shirom A. (2003). Job-related burnout: A review. In Quick C., & Tetrick L. E., (eds), *Handbook of occupational health psychology*. Washington, DC: American Psychological Association 2003. (pp. 245–265).

Shirom A., & Melamed, S. (2006). A comparison of the construct validity of two burnout measures in two groups of professionals. *International Journal of Stress Management, 13(2),* 176–200.

Smout, M., Davies, M., Burns, N., & Christie, A. (2014). Development of the Valuing Questionnaire (VQ). Journal of Contextual Behavioral Science, 3*(3),* 164–172. doi:10.1016/j.jcbs.2014.06.00.
